# Anti-Tumor Effects of Sheep Umbilical Cord Mesenchymal Stem Cells on Melanoma Cells

**DOI:** 10.3390/ijms26010426

**Published:** 2025-01-06

**Authors:** Fengjiao Yue, Yuqing Zhao, Yiting Lv, Songmei Li, Weihai Wang, Yajun Li, Shujie Wang, Chunsheng Wang

**Affiliations:** 1 College of Life Science, Northeast Forestry University, Harbin 150040, China; 15714600516@163.com (F.Y.); 2024122914@nefu.edu.cn (Y.Z.); 2024123013@nefu.edu.cn (Y.L.); 2022122858@nefu.edu.cn (S.L.); 2023113060@nefu.edu.cn (W.W.); liyajun1014@163.com (Y.L.); 2 State Key Laboratory for Animal Disease Control and Prevention, Harbin Veterinary Research Institute, Chinese Academy of Agricultural Sciences, Harbin 150001, China

**Keywords:** mesenchymal stem cells, melanoma, RNA-seq, nude mouse, subcutaneous tumor

## Abstract

Melanoma is among the most common malignancies and has recently exhibited increased resistance to treatments, resulting in a more aggressive disease course. Mesenchymal stem cells (MSCs) secrete cytokines both in vivo and in vitro, which regulate tumor cell signaling pathways and the tumor microenvironment, thereby influencing tumor progression. This study investigates the anti-melanogenesis effects of sheep umbilical cord mesenchymal stem cells (SUCMSCs) to assess their potential application in melanoma treatment. Our findings indicate that, in vitro, SUCMSCs reduce melanin content and tyrosinase activity, inhibit melanoma cell viability, proliferation, migration, and invasion, and promote melanoma cell apoptosis. Subsequent in vivo experiments confirmed that SUCMSCs effectively suppress tumor growth, and histological analysis via HE staining revealed notable differences. Additionally, transcriptome sequencing analysis indicated that the anti-tumor effects were primarily mediated through autophagy, apoptosis, and the TGF-β and NF-κB signaling pathways. The RT-qPCR validation results aligned with the transcriptome data. In summary, SUCMSCs exert anti-melanogenesis effects through the interaction of multiple signaling pathways and cytokines, demonstrating significant potential for melanoma treatment.

## 1. Introduction

Melanoma is a major type of skin cancer and the most malignant form of skin tumor. It is usually caused by a pernicious transformation of a normal mole or pigmented spot. Melanomas are usually characterized by a radial growth phase (RGP) and during the vertical growth phase (VGP), melanomas invade deep into the tissue and are extremely aggressive [1]. VGP melanoma is very difficult to control, with a 5-year survival rate of less than 20%, severely affecting the quality of life of patients, making it one of the deadliest forms of skin cancer. Numerous risk factors for melanoma tumorigenesis have been identified, most of which can be categorized as genetic or environmental [2]. A family history of melanoma is a notable genetic risk factor. The pathogenesis, prognosis, and treatment of melanoma caused by environmental factors are closely related to mutations in genes such as *BRAF*, *CKIT*, *NRAS*, etc. The B-Raf proto-oncogene, a serine-threonine protein kinase, has the most mutations at codon 600, including V600E (80%), V600K (15%), and V600R/M/D/G (5%). Glutamate is the most common substitute for valine [3]. Normally, the BRAF gene is activated only during cell proliferation, but BRAF mutations remain active, activating the downstream MAPK (mitogen-activated protein kinase) signaling pathway and causing uncontrolled cell invasion and proliferation, leading to the rapid progression of melanoma in situ to metastatic malignant melanoma [4]. Over time, melanoma becomes less sensitive to drugs such as cisplatin and targeted inhibitors, reducing treatment efficacy and producing serious side effects. Therefore, there is an urgent need for novel anti-cancer drugs to improve treatment outcomes.

Mesenchymal stem cells (MSCs) are adult stem cells derived from the mesoderm, characterized by self-renewal and multidirectional differentiation potential, and are morphologically like fibroblasts. MSCs can differentiate into various cell types, including adipocytes, chondrocytes, and osteocytes. They are recognized for their hematopoietic support, facilitation of stem cell migration, and immunomodulatory properties [5]. MSCs have garnered considerable interest as a cell-based therapy. Research has demonstrated that MSCs can interact with tumor cells to either promote or inhibit tumor growth and metastasis, positioning MSCs as promising candidates for melanoma treatment. MSCs exhibit tumor tropism and can migrate to both primary and metastatic tumors, irrespective of tumor type or host immune status [6]. On the one hand, MSCs provide a structural framework for anchoring tumor cells [7]; on the other, they secrete numerous chemokines and growth factors in response to tumor stimuli, which can directly differentiate into immune cells and influence the proliferation and metastasis of tumor cells via paracrine signaling [8]. Therefore, MSCs have a promising future in the treatment of tumors.

Previous studies have shown that sheep umbilical cord-derived MSCs can treat conditions such as myelomeningocele (MMC) [9], intervertebral disc degeneration (IVD) [10], and osteoarthritis [11]. Moreover, MSCs have demonstrated therapeutic efficacy against breast cancer [12]. MSCs derived from sheep umbilical cord can be harvested from the umbilical cord tissue, which is typically discarded after lambing, thus avoiding ethical concerns associated with cell acquisition. By contrast, human stem cells often face intense ethical scrutiny and legal restrictions, limiting the scope of research. Furthermore, sheep MSCs offer lower culture and expansion costs, making them more practical for basic research. These cells have been isolated, cultured, expanded, and characterized similarly to human MSCs and have been utilized in studies of human medical applications. As a result, sheep MSCs were selected for investigating their effects on human melanoma cells. Due to their paracrine activity, MSCs can secrete significant amounts of growth factors, cytokines, chemokines, and other bioactive components into the culture medium, referred to as conditioned medium (CM) [13]. Therefore, we used sheep MSC-derived conditioned medium (SUCMSC-CM) in our experiments. It reduces the potential for gene mutations and immunogenicity and provides stability and safety [14,15], which is expected to inhibit the occurrence and progression of melanoma. In preparation for our experiment, we investigated the effects of sheep umbilical cord mesenchymal stem cells (SUCMSCs) on various cancer cell lines, including melanoma (B16, A375), human liver cancer (7721, HEPG2), human neuroblastoma (SY5Y), and human pancreatic cancer (8988T). We found that SUCMSCs exhibited the most pronounced therapeutic effects against B16 and A375 cells. The results from Transwell assays and V-FITC/PI double staining revealed that MSCs could inhibit the invasion of B16 and A375 cells while promoting apoptosis. Further studies were conducted to examine the anti-tumor signaling pathways and target genes of SUCMSCs. The findings indicated that the anti-tumor effects were primarily mediated through autophagy, apoptosis, and the TGF-β and NF-κB signaling pathways. The interactions among multiple signaling pathways and cytokines were confirmed to contribute to the observed anti-tumor effects. This study is anticipated to offer a novel approach to melanoma treatment, potentially improving patient survival rates.

## 2. Results

**Figure 1:**
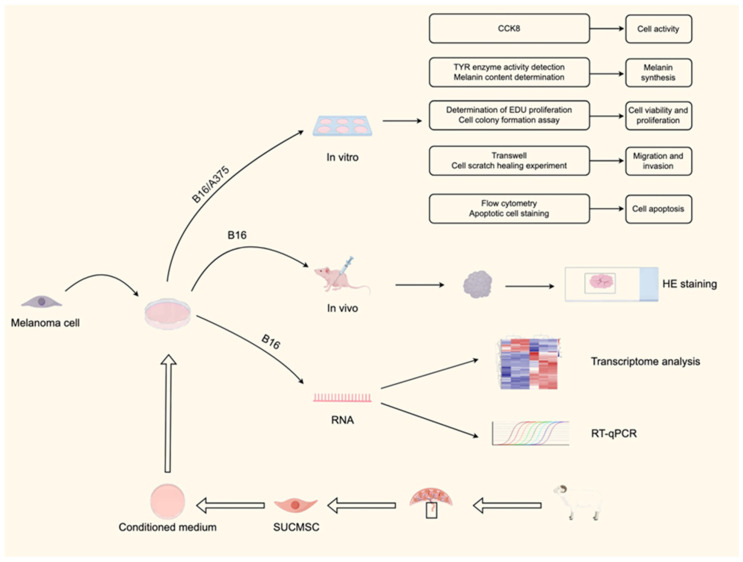
T Flow diagram of the experiment to explore sheep umbilical cord mesenchymal stem cells (SUCMSCs) against melanoma.

This study demonstrated the anti-tumor effect of SUCMSCs on melanoma cells and revealed the molecular mechanism by which SUCMSCs inhibit tumor growth by affecting multiple signaling pathways. The flow chart is shown in Figure 1.

### 2.1. SUCMSCs Affect Micromorphological Characteristics and Inhibit Melanin Synthesis

**Figure 2:**
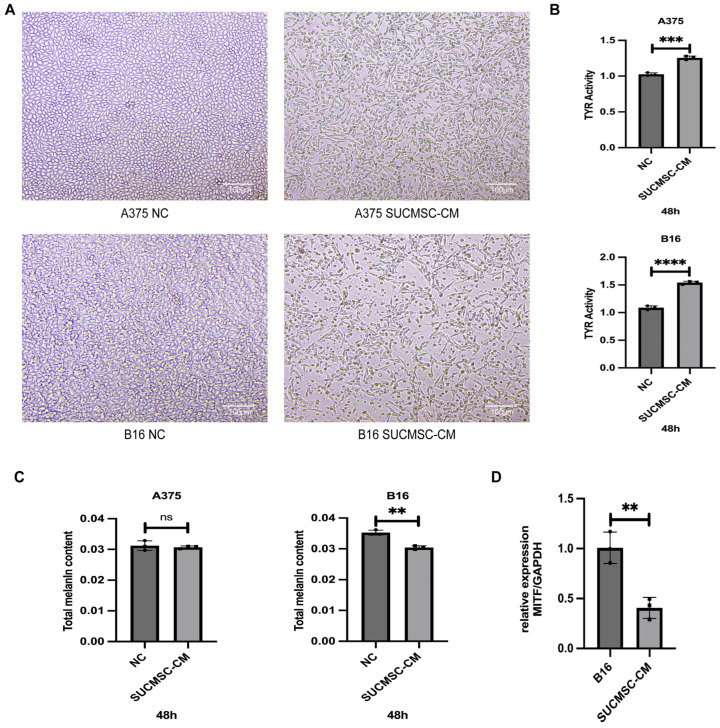
SUCMSCs inhibit melanoma cell viability and melanin synthesis, affecting micromorphological characteristics. (**A**). Morphological changes in melanoma cells following SUCMSC-CM intervention for 48 h. Scale bars = 100 µm. (**B**). The effect of sheep MSC-derived conditioned medium (SUCMSC-CM) on tyrosinase activity in melanoma cells was determined using the L-Dopa oxidation rate assay. (**C**). The total intracellular melanin content was assessed via alkaline lysis. (**D**). qPCR results for melanoma marker. Results are expressed as the mean ± SD, and significance was determined using one-way ANOVA and the Tukey multiple-comparison test. ****, *p* < 0.0001; ***, *p* < 0.001; **, *p* < 0.01; ns, not significant.

Cell morphology analysis revealed that melanoma cells in the control group exhibited an epithelial cell type and were closely arranged. Following treatment with SUCMSC-CM, an increase in the number of dead cells was observed, with intercellular arrangements becoming sparse and the cells appearing shriveled (Figure 2A).

Given that SUCMSCs affected the morphology of melanoma cells, we further investigated their impact on melanin production and tyrosinase (Tyr) expression. Tyrosinase is a key enzyme involved in melanin synthesis. The results from the L-Dopa assay indicated a significant increase in intracellular tyrosinase (TYR) activity in both B16 and A375 cells following SUCMSC treatment (*p* < 0.001) (Figure 2B). Melanin production assays showed a reduction in total intracellular melanin content in B16 cells (*p* < 0.01), while no significant changes were observed in A375 cells (*p* > 0.05) (Figure 2C). The protein encoded by MITF (microphthalmia-associated transcription factor) regulates melanocyte development and melanopoiesis-specific gene transcription. The RT-qPCR results showed upregulation of MITF expression (Figure 2D). These findings suggested that SUCMSCs inhibited melanoma cell viability and melanin synthesis.

### 2.2. SUCMSCs Suppress Melanoma Cell Proliferation

**Figure 3:**
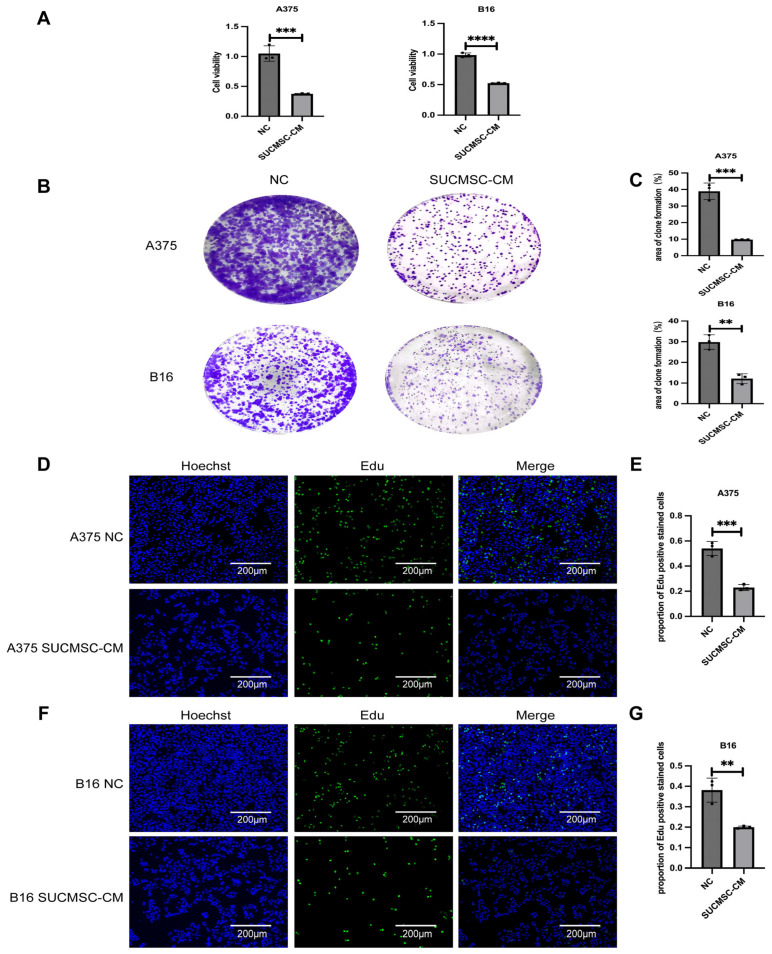
The effects of SUCMSC-CM on A375 melanoma cell viability and proliferation. (**A**). CCK-8 assay analysis of the viability of melanoma cells in the SUCMSC-CM treatment group compared to the control group. (**B**). The cell colony assay was employed to evaluate the impact of SUCMSC-CM on melanoma colony formation. (**C**). Bar graph depicting cell proliferation under various treatment conditions. (**D**). Photomicrographs showing nuclear morphology in treated A375 cells stained for EdU (green) and Hoechst (blue). Scale bars = 200 µm. (**E**). Bar graph illustrating the percentage of A375 nuclei double-labeled for EdU and Hoechst in the SUCMSC-CM treatment group versus the control group. (**F**). Photomicrographs displaying nuclear morphology in treated B16 cells stained for EdU (green) and Hoechst (blue). Scale bars = 200 µm. (**G**). Bar graph showing the percentage of B16 nuclei double-labeled for EdU and Hoechst across treatment conditions. Results are expressed as the mean ± SD, and significance was determined using one-way ANOVA and the Tukey multiple-comparison test. ****, *p* < 0.0001; ***, *p* < 0.001; **, *p* < 0.01.

To further explore the impact of SUCMSCs on melanoma cell proliferation, we employed the CCK-8 assay to measure the in vitro viability of B16 and A375 cells. Following treatment with SUCMSC-CM for 48 h, absorbance values were measured using a microplate reader. The results revealed that SUCMSCs significantly inhibited melanoma cell viability (*p* < 0.001) (Figure 3A).

Colony formation assays revealed significant reductions in the numbers of A375 and B16 cells following SUCMSC-CM treatment (Figure 3B,C). In the B16 group, SUCMSC-CM treatment adversely affected cell proliferation compared to the control group (*p* < 0.001, Figure 3C). Similarly, in the A375 group, SUCMSC-CM treatment led to a more pronounced inhibition of cell proliferation compared to the control group (*p* < 0.01, Figure 3C). In the A375 cell experiments, nuclei were stained blue with HOECHST (Beyotime; Shanghai, China), revealing a marked difference in the population of HOECHST-positive cells, with a reduction in the number of nuclei following SUCMSC-CM treatment (Figure 3D). In the A375 group, SUCMSC-CM treatment significantly reduced cell proliferation compared to the control group (*p* < 0.001, Figure 3E). A similar conclusion was drawn from the data for the B16 group (Figure 3F), confirming that SUCMSCs had an inhibitory effect on melanoma cell proliferation (*p* < 0.001, Figure 3G).

### 2.3. SUCMSCs Inhibit the Migration and Invasion of Melanoma Cells

**Figure 4:**
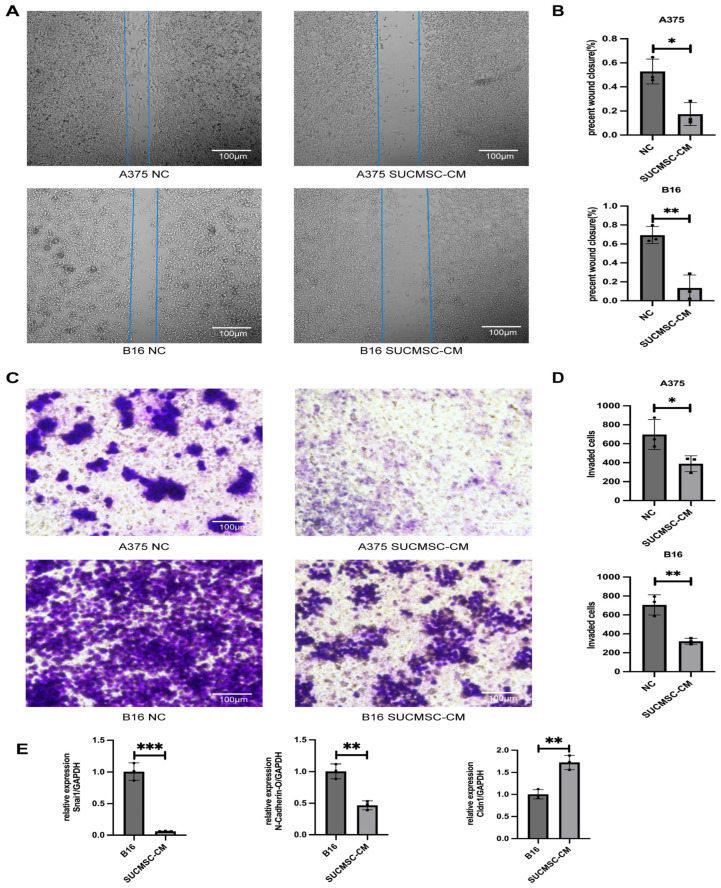
SUCMSC-CM inhibits migration and invasion of A375 melanoma cells. (**A**). Photomicrographs representing A375 monolayers at 0 h and 48 h following mechanical scraping. (**B**). Bar graphs showing the mean percentage of wound closure under different conditions. Scale bars = 100 µm. * *p* < 0.05; ** *p* < 0.01. (**C**). The Transwell assay was conducted to evaluate cell invasion in A375 or B16 cells after treatment with CM or control medium for 48 h. (**D**). Bar graph showing the mean number of A375 or B16 cells present on the lower membrane following 48 h. Scale bars = 100 µm. (**E**). qPCR results for phenotypic switch markers. Results are expressed as the mean ± SD, and significance was determined using one-way ANOVA and the Tukey multiple-comparison test. ***, *p* < 0.001; **, *p* < 0.01; * *p* < 0.05.

Cell migration and invasion are critical aspects of tumorigenicity. To assess the effects of SUCMSCs on melanoma cell migration, we performed a wound healing assay. After 48 h of SUCMSC-CM treatment, a significant reduction in the number of migrating melanoma cells was observed compared to the control group (*p* < 0.05) (Figure 4A,B). We further evaluated the effect of SUCMSCs on melanoma cell invasion using a Transwell assay. The results showed a marked reduction in the number of cells migrating to the lower compartment through the Matrigel following SUCMSC-CM treatment (*p* < 0.01) (Figure 4C,D). Snai1 (snail family transcriptional repressor 1) is a zinc finger transcription factor that regulates tumor growth and metastasis. N-Cadherin is a class of cell–cell adhesion molecules, mainly distributed in various epithelial tissues, mediating cell-to-cell adhesion. RT-qPCR showed that both Snai1 and N-Cadherin expression were downregulated (Figure 4E). Cldn1 (claudin 1) is an important intercellular connexin, which is a membrane protein that forms tight junction bands on cell membranes. RT-qPCR showed that Cldn1 expression was upregulated (Figure 4E). Taken together, our results suggested that SUCMSCs inhibited the migration and invasion of melanoma cells.

### 2.4. SUCMSCs Induce Apoptosis in Melanoma Cells

**Figure 5:**
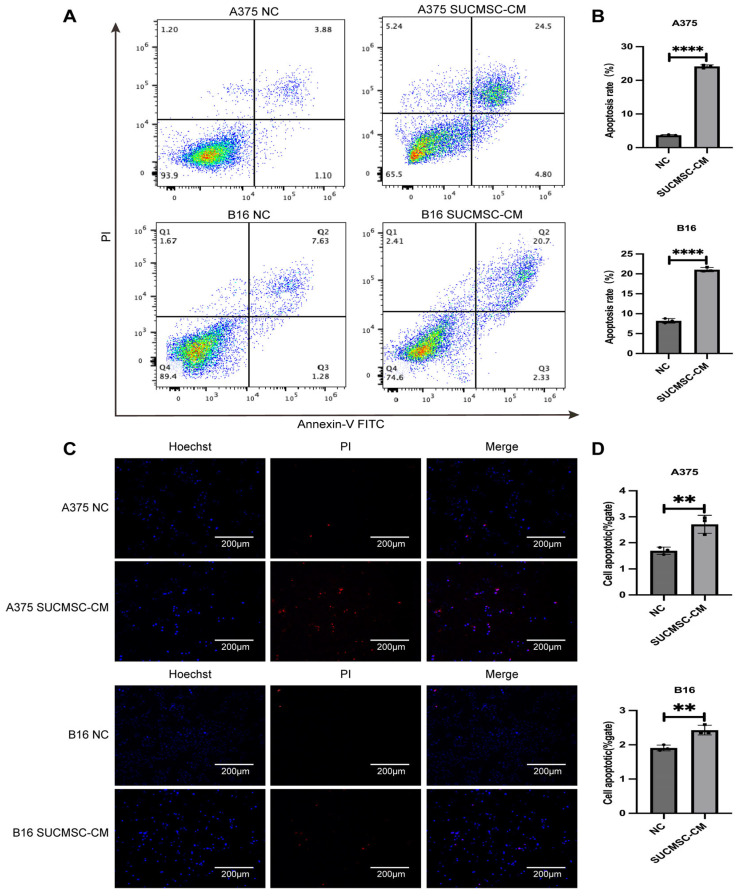
SUCMSCs induce cell apoptosis. Annexin V/PI double staining was employed to assess the effect of SUCMSC-CM on melanoma cell apoptosis. (**A**). Representative plots from flow cytometry analysis of Annexin V-FITC and PI-stained A375 melanoma cells, indicating an increase in the number of apoptotic cells after SUCMSC-CM treatment. (**B**). Bar graphs displaying the mean percentage of PI-stained A375 or B16 nuclei that were Annexin V-FITC positive. (**C**). The Hoechst 33342/PI staining assay was used to determine apoptosis in melanoma cells. (**D**). Bar graphs showing the mean ± standard deviation for three independent experiments. Scale bars = 100 µm. Results are expressed as the mean ± SD, and significance was determined using one-way ANOVA and the Tukey multiple-comparison test. ****, *p* < 0.0001; **, *p* < 0.01.

To evaluate the effect of SUCMSC-CM on apoptosis in B16 and A375 cells, we used Annexin V-FITC/PI staining and flow cytometry analysis. Flow cytometry analysis showed that, after SUCMSC-CM treatment, we observed an increase in the number of apoptotic cells, especially in advanced apoptosis (Figure 5A). The percentages of A375 and B16 cells with apoptosis were significantly higher in the treatment groups compared to the control groups (*p* < 0.001, Figure 5B). In addition, the Hoechst 33342/PI staining assay showed that SUCMSC-CM treatment promoted apoptosis compared to untreated controls (*p* < 0.01, Figure 5C,D). These findings suggested that SUCMSCs effectively promoted apoptosis in melanoma cells.

### 2.5. SUCMSCs Inhibit Melanoma Tumor Growth in Nude Mouse

**Figure 6:**
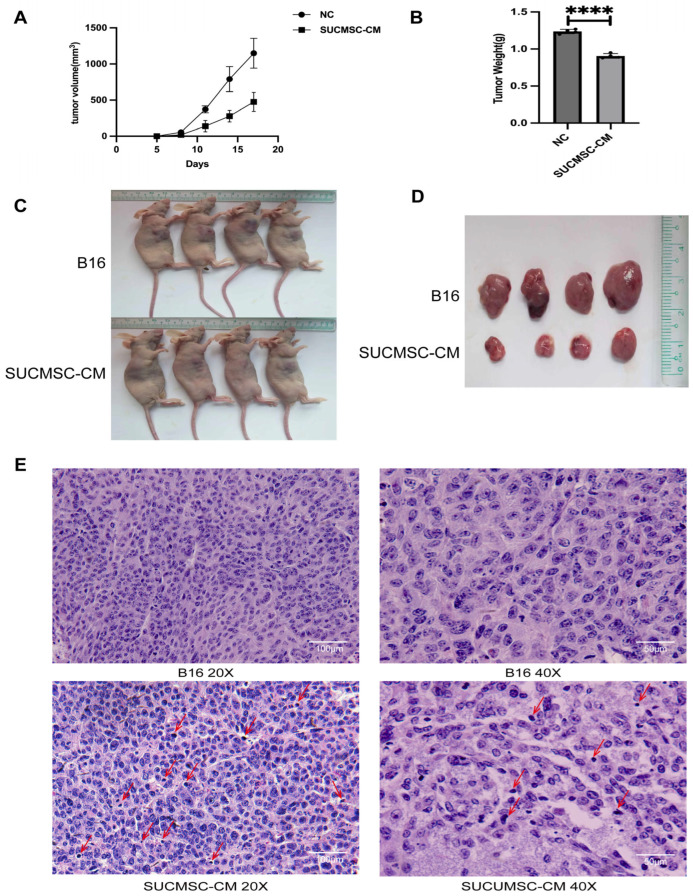
SUCMSC-CM inhibits malignant progression of melanoma. (**A**). Growth curves of tumors showing the average volume of eight tumors in the SUCMSC-CM treatment group versus the control group over 5-17 days. (**B**). Bar graph displaying mean tumor weights on day 17 across treatments. (**C**). Tumor images of nude mice 17 days after the injection of B16 cells in either SUCMSC-CM or control medium. (**D**). Tumors excised at day 17 from nude mice injected with B16 cells in SUCMSC-CM or control medium. (**E**). Hematoxylin-eosin staining showing representative tumor histopathology in the SUCMSC-CM treatment group and the control group. Scale bars = 100 µm. Results are expressed as the mean ± SD, and significance was determined using one-way ANOVA and the Tukey multiple-comparison test. ****, *p* < 0.0001.

We then detected melanoma tumor formation in a nude mouse transplant model, continuously monitoring the tumor volume every 3 days, and removing and weighing the tumor on day 17 (*n* = 8, *p* < 0.05). First of all, a significant reduction in tumor volume was observed in the SUCMSC-CM-treated group (Figure 6A,C,D). Tumor weight was also lower in the SUCMSC-CM-treated group compared to the control group (*p* < 0.05, Figure 6B). Histological analysis of tumor sections, stained with hematoxylin and eosin (HE), revealed a reduction in tumor cell number (fewer blue nuclei) in the SUCMSC-CM group. The intercellular space was increased, with more connective tissue, and an abundance of apoptotic bodies was observed (indicated by red arrows) (Figure 6E). These results suggested that SUCMSC-CM inhibited melanoma tumor growth in vivo. In conclusion, SUCMSC-CM effectively suppressed the malignant progression of melanoma.

### 2.6. SUCMSCs Inhibit Melanoma Growth Through Multiple Pathways

**Figure 7:**
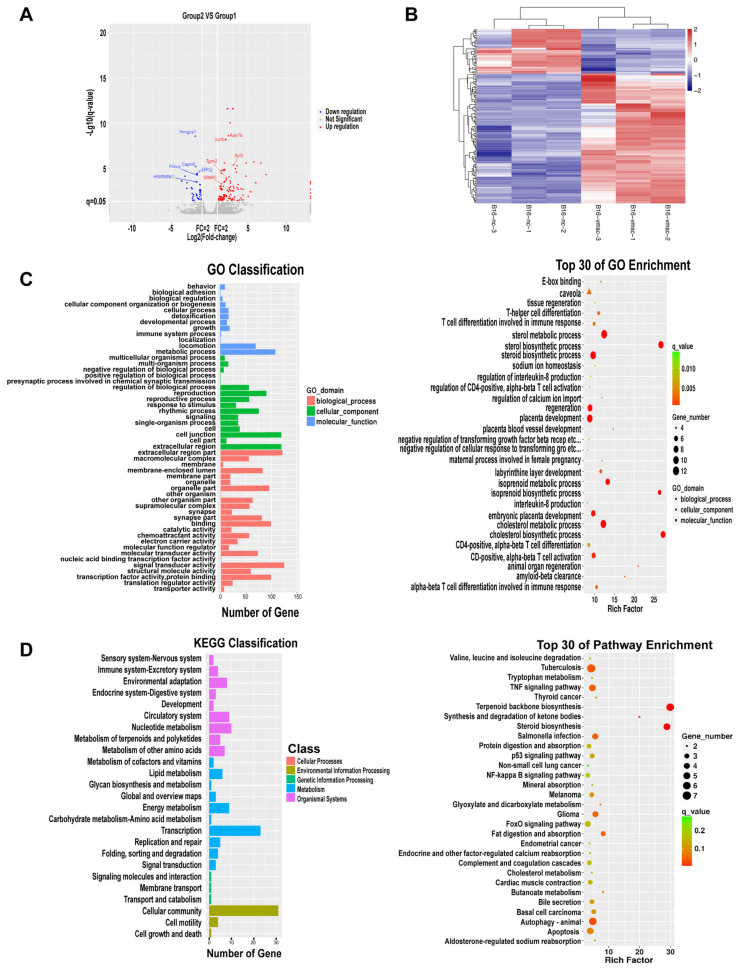
RNA sequencing (RNA-seq) analysis revealed the underlying mechanism of melanoma inhibition by SUCMSCs. (**A**). Volcano plot of differentially expressed genes. (**B**). Heat map of differentially expressed genes. (**C**). Gene Ontology (GO) enrichment analysis of differentially expressed genes indicated that DEGs were most enriched in steroid and sterol biosynthesis, cholesterol metabolism, and regeneration. (**D**). KEGG enrichment analysis demonstrated that SUCMSCs may promote apoptosis in B16 cells by enhancing autophagy, as well as inhibiting the TGF-β and NF-κB signaling pathways.

In the volcano plot of differential expression, red indicates upregulated differentially expressed genes (DEGs) and blue indicates downregulated DEGs (Figure 7A). After obtaining the transcriptome data, we constructed a digital gene expression matrix and identified significant changes in gene expression following SUCMSC-CM treatment (Figure 7B). Gene Ontology (GO) enrichment analysis and KEGG pathway analysis were performed on the DEGs. GO analysis revealed three categories: biological processes, cellular components, and molecular functions. The results indicated that DEGs were most enriched in biological processes such as biological regulation, cellular processes, and monobiotic processes. In terms of cellular components, DEGs were primarily associated with cellular structures. Regarding molecular functions, most DEGs exhibited significant binding and catalytic activities. The bubble plots of the GO enrichment analysis demonstrated that DEGs were most enriched in steroid and sterol biosynthesis, cholesterol metabolism, and regeneration (Figure 7C). KEGG pathway analysis showed that the differentially expressed genes were mainly enriched in five pathways: the digestive system, environmental information processing, genetic information processing, metabolism, and biological systems. The bubble plots of the KEGG enrichment analysis suggested that SUCMSCs may promote apoptosis in B16 cells by enhancing autophagy, inducing apoptosis, and inhibiting the TGF-β and NF-κB signaling pathways (Figure 7D).

**Figure 8:**
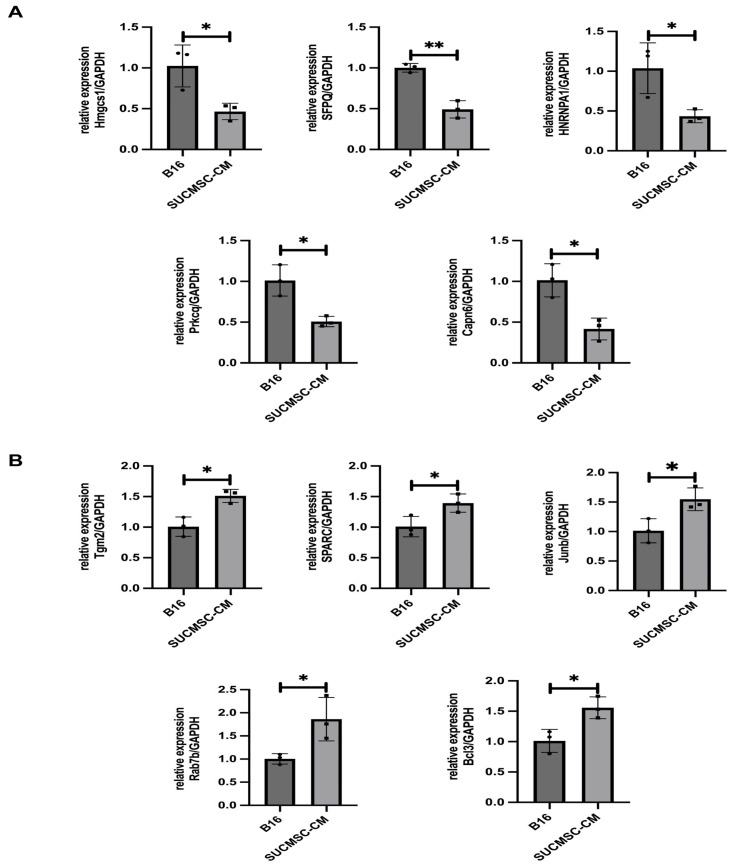
Validation of transcriptome results by RT-qPCR. (**A**). qPCR results for downregulated genes. (**B**). qPCR results for upregulated genes. Results are expressed as the mean ± SD, and significance was determined using one-way ANOVA and the Tukey multiple-comparison test. **, *p* < 0.01; * *p* < 0.05.

Based on these pathways, we selected 10 genes for validation using RT-qPCR. The validation results revealed that the gene expression of Hmgcs1 (3-hydroxy-3-methylglutaryl-CoA synthase 1), Capn6 (calpain 6), HNRNPA1 (heterogeneous nuclear ribonucleoprotein A1), SFPQ (splicing factor proline and glutamine rich), and Prkcq (protein kinase C theta) was significantly decreased in the SUCMSC-CM-treated group, while Bcl3 (BCL3 transcription coactivator), Tgm2 (transglutaminase 2), Rab7b (Rab7b, member of RAS oncogene family), Junb (jun B proto-oncogene), and SPARC (secreted acidic cysteine rich glycoprotein) expression was significantly increased in the same group. These results indicated that SUCMSCs promoted autophagy and apoptosis while inhibiting the TGF-β and NF-κB signaling pathways by downregulating Hmgcs1, Capn6, HNRNPA1, SFPQ, and Prkcq and upregulating Bcl3, Tgm2, Rab7b, Junb, and SPARC (Figure 8). Consequently, the progression of melanoma growth was inhibited (Figure 8).

## 3. Discussion

Melanoma is a tumor with a low incidence but a high degree of malignancy, resulting from the malignant transformation of melanocytes [16]. Previous studies have demonstrated that MSCs exhibit potential therapeutic effects against various tumor-related diseases and cancers, providing new avenues for the treatment of malignancies such as melanoma. For instance, He et al. showed that MSCs can inhibit tumor progression by downregulating the Stat3 signaling pathway [17]. Similarly, Aravindhan et al. found that MSCs preferentially migrate into tumor tissues. Furthermore, the interaction of MSCs with the tumor microenvironment may be advantageous for cancer treatment [18]. However, the potential tumorigenicity of stem cell therapy must be considered, as it poses significant risks. Additionally, MSCs exhibit paracrine activity, allowing them to secrete substantial quantities of growth factors, cytokines, chemokines, and other bioactive components into their culture medium, referred to as conditioned medium (CM) [13]. Zhou and colleagues showed that human placenta-derived MSC extracellular vesicles (hPMSC-EVs) inhibit tumor cell proliferation and migration, as well as breast cancer cell growth, through an indirect anti-angiogenic mechanism [19]. These findings suggested that MSC-conditioned medium may have inhibitory effects against tumor development. Therefore, we utilized MSC-derived conditioned media in our experiments. This cell-free therapy offers stability and safety by minimizing potential gene mutagenicity and immunogenicity [14,15] and is anticipated to inhibit the occurrence and progression of melanoma. In this study, we prepared conditioned medium from sheep umbilical cord MSCs to investigate its effects on melanoma.

Initially, we conducted an in vitro experiment. SUCMSC-CM affected the morphology of melanoma cells under a light microscope. The A375 and B16 cell lines, which exhibited fusiform and epithelioid features, showed a significant reduction in cell number after 48 h of conditioned medium treatment (Figure 2A), accompanied by increased tyrosinase activity and decreased melanin content (Figure 2C). Additionally, the expression of melanoma markers decreased in the SUCMSC-CM treatment group (Figure 2D). Furthermore, after conditioned medium treatment, the number of proliferating melanoma cells was significantly reduced and the viability of the cells decreased (Figure 3). Given that melanoma is a malignant tumor, the degree of malignancy is closely related to the metastasis and invasion of melanoma cells [20]. The results from the wound healing assay and Transwell assay demonstrated reductions in metastatic and invasive potential for both B16 and A375 cells (Figure 4A,C). Several markers can serve as indicators of melanoma phenotype changes, such as Snai1 [21], N-cadherin [22], and Cldn1 [23]. We examined the expression of these differentiation and invasion markers, and the qPCR results showed that the migration ability of the SUCMSC-CM treatment group was reduced. Moreover, the Transwell assay revealed a decrease in the invasive capacity of these cell lines (Figure 4C). These findings are consistent with those of X. Yao et al., who demonstrated that BM-MSCs inhibit the invasion, migration, proliferation, and deterioration of pancreatic cancer cells [24]. Furthermore, other studies have suggested that MSCs can induce apoptosis by arresting the cell cycle or altering cell cycle distribution [25,26]. Next, we hypothesized that MSC conditioned medium might promote the apoptosis of melanoma cells. Flow cytometry and a necrosis detection kit were used to verify the results, which showed that the number of apoptotic cells increased in SUCMSC-CM treatment group (Figure 5A–D). However, MSCs home to the tumor site at the later stage of tumor development, and tumor cells and other cells exert certain effects on them, which endows MSCs with the properties of promoting tumorigenesis, development, and metastasis [27]. For instance, Xu et al. demonstrated that MSCs promote colorectal cancer (CRC) cell progression and metastasis by secreting CCL7 and TGF-β, as well as regulating CXCL5 expression in CRC cells to promote proliferation and metastasis [28]. This phenomenon may be attributed to the dose of MSCs, as the injection of excessive MSCs carries the risk of promoting tumor growth. Zhang et al. found that a small number of MSCs had an anti-tumor effect in mice with lung metastasis of melanoma, while the injection of more than 10⁵ cells promoted tumor growth [29]. The tumor weight and volume data and HE results showed that the tumor size of the mice injected with SUCMSC-CM-treated melanoma cells was significantly smaller than that of the control group, further confirming that SUCMSC-CM could effectively inhibit the growth of melanoma in vivo.

We also explored the potential mechanism by which SUCMSC-CM inhibited melanoma progression. Transcriptome analysis of upregulated and downregulated genes (Figure 7A,B), along with GO enrichment and KEGG pathway analysis, revealed that SUCMSC-CM could inhibit melanoma growth through the NF-κB, TGF-β, autophagy, and apoptosis pathways. RT-qPCR validation of potential regulatory factors involved in the inhibition of B16 cells was also performed. The potential regulatory factors of SUCMSCs inhibiting B16 cells were screened and verified by RT-qPCR. The five downregulated genes we identified are involved in the NF-κB, TGF-β, and apoptosis pathways. Downregulation of hnRNPA1 inhibits the NF-κB pathway, leading to reduced telomerase activation, unsustainable telomere length, and inhibition of tumor development [30,31]. Additionally, downregulation of HMGCS1 and Prkcq inhibits cancer cell proliferation, migration, and invasion [32,33,34]. Downregulation of SFPQ blocks TGF-β signaling, inhibiting tumor growth [35]. Furthermore, downregulation of Capn6 promotes tumor cell apoptosis and inhibits angiogenesis, thereby reducing tumor formation [36]. The five upregulated genes identified are associated with the NF-κB, TGF-β, and autophagy pathways. Upregulation of Tgm2 and Rab7b promotes tumor cell autophagy [37,38,39,40]. Upregulation of Junb and Bcl3 induces the expression of NF-κB-dependent proinflammatory cytokines, thereby inhibiting tumor growth [41,42,43]. Lastly, upregulation of SPARC inhibits TGF-β signaling, which also reduces tumor growth [44,45]. Therefore, the RT-qPCR results showed that the expression changes of key genes were consistent with the transcriptome analysis. In conclusion, we speculate that SUCMSCs inhibit melanoma progression by promoting autophagy and apoptosis while inhibiting the TGF-β and NF-κB signaling pathways.

Despite the evidence from in vitro and animal experiments indicating that SUCMSC-CM effectively inhibits tumor growth, several limitations and challenges remain. MSCs can regulate adaptive and innate immunity through the secretion of cytokines or via cell-to-cell contact, and they exhibit low immunogenicity [46]. However, the conditioned medium contains various growth factors, cytokines, and other bioactive substances [13], and the precise mechanisms of action are not fully understood. Further research is required to gain deeper insights into the molecular mechanisms underlying MSC-mediated tumor growth inhibition, with the aim of optimizing treatment protocols and enhancing efficacy.

## 4. Materials and Methods

### 4.1. Cell Culture

Stem cells were isolated from Wharton’s jelly in sheep umbilical cord tissue and the expression of surface markers (CD14, CD34, CD44, CD45, and CD105) in sheep umbilical MSCs was analyzed by flow cytometry [47]. After thawing in a pre-warmed 37 °C water bath, SUCMSCs were cultured in DMEM/F-12 medium (containing 1% 100 U/mL penicillin-streptomycin) supplemented with 10% fetal bovine serum (FBS). B16 and A375 cells (purchased from the Cell Center, Chinese Academy of Medical Sciences, Beijing, China) were maintained in H-DMEM supplemented with 10% FBS and containing 1% penicillin-streptomycin. The medium was replaced every two days, and once 80% confluency was reached, adherent cells were harvested using trypsinization and expanded over 3–4 passages for experiments.

### 4.2. Preparation of SUCMSC-CM

Primary SUCMSCs (isolated and cultured by the Laboratory of Animal Developmental Biology, Northeast Forestry University) were cultured in complete medium comprising DMEM/Ham’s F-12 (1:1), L-glutamine, 10% fetal bovine serum (FBS; Gibco, Shanghai, China), and 1% penicillin-streptomycin (100 U/mL; Gibco, Shanghai, China). Cells were transferred to 10 cm dishes (Corning, Shanghai, China) and maintained in a 37 °C, 5% CO_2_ incubator (Thermo Fisher, Shanghai, China). When SUCMSCs reached 80% confluency, the medium was replaced with 5 mL of serum-free medium containing 1% penicillin-streptomycin (100 U/mL). After 48 h, the conditioned medium (CM) was collected, centrifuged at 1500 rpm for 5 min, filtered using a 0.45 μm Millex-HP filter (Millipore, Shanghai, China) to generate a 1× concentration of SUCMSC conditioned medium (SUCMSC-CM), and stored at 4 °C.

The following summarizes the number of melanoma cells and volumes of conditioned medium used in each experiment:

CCK8 and TYR assays: 2 × 10^3^ cells and 100 μL of conditioned medium;
EDU and cell scratch assays: 2 × 10^5^ cells and 1 mL of conditioned medium;
Colony formation assay: 5 × 10^3^ cells and 2.5 mL of conditioned medium;
Transwell assay: 5 × 10^4^ cells and 1 mL of conditioned medium;
Apoptosis assay: 1 × 10^5^ cells and 500 μL of conditioned medium;
Tumor formation assay: 2 × 10^5^ B16 cells and 500 μL of conditioned medium;
Flow cytometry assay: 5 × 10^5^ cells and 2.5 mL of conditioned medium.

### 4.3. CCK8 Cytotoxicity Assay

A total of 2 × 10^3^ melanoma cells per well were cultured in 96-well plates, and three double wells were set up and incubated in complete medium for 24 h, after which the medium was changed to SUCMSC-CM (the control group was SFM medium) and the cells were incubated for another 48 h. The assay was the cell counting kit-8 (CCK-8, Targetmol, Shanghai, China). A 10 μL volume of CCK-8 solution was added per well and cells were incubated at 37 °C for 3 h, according to the manufacturer’s instructions. The absorbance of each well was measured using a microplate reader at a wavelength of 450 nm.

### 4.4. TYR Enzyme Activity Detection

Melanoma cells were seeded into 96-well plates at 2 × 10^3^ per well, another 5 wells were taken as a blank zeroing group, and the cells were cultured in an incubator for 24 h. The original medium was discarded, serum-free DMEM medium was added to the control group, conditioned medium was added to the experimental group, 5 replicates were set up in each group, the supernatant was removed after 48 h of incubator culture, 1% Triton-X100 was added to each well, and the cells were completely ruptured by melting at room temperature. The samples were pre-warmed at 37 °C for 5 min, 0.5% DOPA was added, the samples were shaken at 37 °C for 2 h, and the absorbance was measured at 490 nm.

(1) $$\begin{array}{cc} TYR\;activity\;= & (A\;experimental\;group-A\;cell\\ & -\;free\;group)/(A\;blank\;control\;group-A\;cell-free) \end{array}$$

### 4.5. Melanin Content Determination

To confirm the changes in melanin content, melanocytes were cultured in an incubator at 37 °C and 5% CO for 24 h, trypsinized, centrifuged at 1000 rpm for 5 min, and pelleted in 1 Mol NaOH containing DMSO for 1 h at 80 °C. Absorbance was measured at 475 nm using a microplate reader.

### 4.6. Determination of EDU Proliferation

The EdU Cell Proliferation Kit (Beyotime, Shanghai, China) was used to assess cell proliferation. SUCMSC-CM-treated and control (SFM-treated) cells (2 × 10^5^ cells/well) were cultured in 12-well plates (Corning, Shanghai, China) for 48 h, followed by incubation with EdU solution for 2 h at 37 °C, according to the manufacturer’s protocol. Cells were then fixed with 4% paraformaldehyde for 15 min, permeabilized with 0.3% Triton X-100 for 15 min, and treated with 200 µL of Click reaction solution for 30 min. Nuclei were counterstained with Hoechst 33342, and fluorescence imaging was used to detect cell proliferation.

### 4.7. Cell Colony Formation Assay

For the cell colony formation assay, A357 (ATCC, Beijing, China) and B16 melanoma cells in the logarithmic growth phase were seeded into 6-well plates at a density of 5 × 10^3^ cells per well. Cells were incubated at 37 °C with 5% CO_2_. The control group was treated with DMEM/F12 basal medium, while the experimental group received SUCMSC-CM. Each group included three replicates. After fixation with 4% paraformaldehyde, cells were stained with 0.1% crystal violet, allowed to dry, photographed, and counted.

### 4.8. Cell Scratch Healing Experiment

In the cell scratch healing experiment, B16 and A375 melanoma cells were cultured in 12-well plates until confluent and then scratched with a 200 μL pipette tip perpendicular to the plate with uniform force to form a mechanical wound. Cells were cultured in conditioned medium for 48 h. An inverted microscope equipped with a camera was used to take brightfield images.

### 4.9. Transwell

Prepare 50 μL of Matrigel gel on ice: 150 μL of serum-free medium was used to make a coating solution, which was coated on the upper culture chamber. Then, 50,000 cells were seeded onto the membrane of the upper chamber in 300 µL medium and 500 µL Dulbecco’s Modified Eagle Medium containing 10% fetal bovine serum was added to the lower chamber (3 well replicates per group). After 72 h of incubation at 37 °C in humidified air containing 5% CO_2_, the lower part of the membrane was fixed with 4% paraformaldehyde for 10 min at room temperature, washed with PBS, and non-migrated cells were removed from the upper part of the membrane with a cotton swab. Staining with crystal violet (Beyotime, Shanghai, China) was performed for 30 min at room temperature, following three PBS washes, and the cells on the submembrane surface were counted in different areas using a microscope.

### 4.10. Flow Cytometry

Melanoma cells were treated with SUCMSC-CM or control medium for 48 h. Cells were harvested and washed twice with PBS and then treated using the Annexin V-FITC/PI Flow Cytometric Assay Kit (Beyotime, Shanghai, China), following the manufacturer’s instructions. A total of 5 × 10⁵ cells were detected.

### 4.11. Apoptotic Cell Staining

The apoptotic cell staining kit (Beyotime, Shanghai, China) was used to detect apoptosis. B16 cells and A375 cells in good condition were cultured at 1 × 10^5^ cells per well in 24-well plates, and each group was set up with 5 wells. A 500 μL volume of SUCMSC-CM was added to three wells in each group, while 2% DMEM was added to the remaining two wells as a control. After 48 h of incubation, 1 mL of cell staining buffer and 5 μL of Hoechst staining solution were added, and then 5 μL of PI staining solution was added and mixed well. The cells were washed once with PBS and then observed under a fluorescence microscope.

### 4.12. RNA Sequencing and Analysis

RNA sequencing was performed under the conditions that had already been determined. RNA was extracted from B16 cells and SUCMSC-CM-treated B16 cells (*n* = 4/group) using TRIzol reagent (Ambion, Austin, TX, USA). Next-generation sequencing (NGS) technology was used to sequence cDNA using high-throughput sequencers (Illumina Hiseq 2000/2500, Miseq). The quality of sequencing sequences was evaluated using the Q value of sequencing quality and GC content. Low-quality read fragments and linker subsequences were removed. The spliced mapping algorithm of Hisat2 (version: 2.0.4) was used to perform genome mapping on the preprocessed reads. The level of gene expression was estimated by the number of reads mapped to the gene region, counted using Stringtie (version: 1.3.0), and then normalized to FPKM values for subsequent analysis. edgeR was used for inter-sample differential gene analysis, and the differential gene screening conditions were a q-value ≤ 0.05 and fold-change ≥ 2. This was followed by GO analysis and KEGG analysis (gene set enrichment analysis). All data were provided by ShangHai Biotechnology Corporation(SHBIO, Shanghai, China).

### 4.13. Nude Mouse Tumorigenesis Experiment

Eight nude mice (5~6 weeks old) were purchased from the Laboratory Animal Center of the Chinese Academy of Medical Sciences (Beijing, China). The mice were housed in specific pathogen-free (SPF)-grade animal rooms in individually ventilated cages (IVCs), with no more than three animals per cage. The air cleanliness was maintained at a level of <10,000 particles per cubic foot, noise levels were maintained at ≤60 decibels, temperature was kept at 20–27 °C, and the humidity level was between 40 and 70%. The mice were maintained under a 12-h light/dark cycle and were given free access to water and food. The experimental animals were allowed to adapt to the animal room for 1 week before the start of the experiment. Due to their lack of immune system, nude mice are more susceptible to engraftment of exogenous cells or tissues. The present study received approval from the Animal Ethics Committee of Northeast Forestry University (protocol code 2021001, November 2020). According to the method of Miao He et al. [48], the B16 cells treated with 100 μl of 2 × 10^5^ SUCMSC-CM and the B16 cells cultured in complete medium were injected respectively into the axilla of four mice as experimental and another four mice as control groups. The mice were weighed every 3 days, and tumor width (*W*) and length (*L*) were measured with calipers. Tumor volume was estimated according to the standard formula $V=1/2\times L\times w^{2}\;$ (*n* = 8). On the 17th day after inoculation, the mouse tumor body exceeded 100 mm and the animal was sacrificed by the method of cervical dislocation, and the tumor tissue was removed. In order to compare the differences in tumor tissue structure and cell composition between the groups, the appropriate size sections were fixed with 4% PFA for HE staining, and the other part was stored in a −80 °C freezer for later use.

### 4.14. HE Staining

Melanomalous tumors of nude mice were fixed in 4% paraformaldehyde and embedded in paraffin. The paraffin-embedded tissue was cut into 8 μm sections, dehydrated in ethanol, stained with hematoxylin and eosin, and finally visualized dropwise with neutral resin. Pictures were obtained using a pathology slide scanner.

### 4.15. Real-Time PCR (qRT-PCR)

Primers were provided by GENEWIZ Company (Suzhoou, China). Melanoma cells were collected, and total RNA was extracted with TRIzol^®^ reagent (Ambion, Austin, TX, USA). The RNA concentration and quality were checked using a NanoDrop instrument. Total RNA was transcribed into cDNA using a reverse transcription system for 5 min at 50 °C and 2 min at 85 °C, respectively. Using the ABI 7500 Fast real-time PCR system (Applied Biosystems, Foster, CA, USA), the reaction system was 20 μL, and the expression level of the target gene was calculated using the 2^−△△Ct^ method with the GAPDH gene used as a reference. The sequences of all primers used for RT-qPCR in this study are shown in Table 1.

### 4.16. Statistical Analyses

The data presented represent the average value ± standard deviation from a minimum of three biological replicates. The data exhibiting a normal distribution were subjected to statistical analysis using *t*-tests, one-way ANOVA, or two-way ANOVA, followed by Tukey’s multiple comparison test, and the results are displayed as the mean ± SD and *p*-value < 0.05. FCS files were exported to FlowJo for subsequent analysis and plot visualization. Data values were imported into Graphpad Prism 9.5 for statistical analysis and graphical representation.

## 5. Conclusions

In conclusion, our study reveals that SUCMSCs exhibit significant anti-cancer effects, offer new treatment strategies for melanoma. The in vitro experiments showed that SUCMSCs can reduce the number of melanoma cells, alter cell morphology, and increase the rate of apoptosis. Tumor formation experiments in nude mice showed that conditioned medium from SUCMSCs significantly reduced tumor volume and weight. Transcriptome sequencing analysis indicated that the anti-cancer effects of SUCMSCs were mainly mediated by autophagy, apoptosis, and the TGF-β and NF-κB signaling pathways, and the results of RT-qPCR were consistent with the transcriptome. In conclusion, the use of SUCMSCs for melanoma treatment holds significant scientific importance, marking it as a potentially valuable strategy in the fight against melanoma.

## Figures and Tables

**Table 1:** List of primers for quantitative PCR detection.

Gene	Forward Primer	Reverse Primer	Length
*Hmgcs1*	AACTGGTGCAGAAATCTCTAGC	GGTTGAATAGCTCAGAACTAGCC	180
*Capn6*	GCGTCCACAGGACATTTCTGATG	GGATCCCATTCCTGATCCTTGTG	177
*HNRNPA1*	GAAACAACCGACGAGAGTCTG	TGTGTGGTCTTGCATTCATGG	163
*SFPQ*	GATCTACAGGGAAAGGCATTGTTG	GATACATTGGATTCTTCTGGGCA	186
*Prkcq*	GAGATGCCGCAAGAACAATGG	ACACTTGACATGGTGGACTTTG	196
*Bcl3*	GAGAGCAGCAGTCGTCTCAG	GGCAGGTGTAGATGTTGTGG	138
*Tgm2*	CCGAGTGGGGGACAGTATGAGC	GGCCCCGCACCTTGATGAG	263
*Rab7b*	TCGAGGAATACCAGACCACAC	ACAGCCATCGGAACCTTTGTA	152
*Junb*	CTATCGGGGTCTCAAGGGTC	CTGTTGGGGACGATCAAGC	147
*SPARC*	TGGGAGAATTTGAGGACGGTG	GAGTCGAAGGTCTTGTTGTCAT	208
*MITF*	GAGTCATGCAGTCCGAATCG	GTAAGCGGGACCCTAAATG	235
*Snail*	TCGAGGAATACCAGACCACAC	TGAATACTGAGGGGTAGGAGGC	210
*N-cadherin*	GCATCTCTGGATGCCCTTCC	CGTGGAGAAAGTGGAGAACATG	133
*Cldn1*	GACTGTGGATGTCCTGCGT	CCAATTTCGAGGGTAGCCTCTGG	175

## Data Availability

All data generated and analyzed during this study are included in this published article. The raw datasets used and/or analyzed during the current study are available from the corresponding author on reasonable request.

## Abbreviations

MSCs	Mesenchymal stem cells
SUCMSCs	Sheep umbilical cord MSCs
CM	Conditioned medium
SUCMSC-CM	Conditioned medium of sheep umbilical cord mesenchymal stem cells
SPF	Specific pathogen-free
IVCs	Individually ventilated cages
Hmgcs1	3-Hydroxy-3-methylglutaryl-CoA synthase 1
Capn 6	Calpain 6
HNRNPA1	Heterogeneous nuclear ribonucleoprotein A1
SFPQ	Splicing factor proline- and glutamine-rich
Prkcq	Protein kinase C theta
Bcl3	BCL3 transcription coactivator
Tgm2	Transglutaminase 2
Rab7b	Rab7b, member RAS oncogene family
Junb	Jun B proto-oncogene
SPARC	Secreted acidic cysteine rich glycoprotein
MITF	Microphthalmia-associated transcription factor
Snai1	Snail family transcriptional repressor 1
Cldn1	Claudin 1
